# The international dataset on the association between Langerhans Cell Histiocytosis and other malignancies

**DOI:** 10.1016/j.dib.2022.108604

**Published:** 2022-09-15

**Authors:** Francesca Bagnasco, Stefanie-Yvonne Zimmermann, Rudolph Maarten Egeler, Vasanta Rao Nanduri, Bruna Cammarata, Jean Donadieu, Thomas Lehrnbecher, Riccardo Haupt

**Affiliations:** a Epidemiology and Biostatistics, Scientific Directorate, IRCCS Istituto Giannina Gaslini, Genova, Italy; b Division of Pediatric Hematology and Oncology, Hospital for Children and Adolescents, Johann Wolfgang Goethe-University, Frankfurt, Germany; c Princess Maxima Center, The Netherlands; d Department of Paediatrics, Watford General Hospital, Watford, United Kingdom; e DOPO Clinic, Division of Pediatric Hematology and Oncology, IRCCS Istituto Giannina Gaslini, Genova, Italy; f Service d'Hémato-oncologie Pédiatrique, Hôpital Armand Trousseau Aphp, Paris, France

**Keywords:** Cancer predisposition, Cancer genetics, Second neoplasms, Langerhans Cell Histiocytosis

## Abstract

This article presents the international dataset of cases in which the association of Langerhans cell Histiocytosis (LCH) with other malignancies (AM) was documented occurring at any age before, concurrently or after LCH. These data are mostly derived from previously published manuscripts or from completed case report forms (CRFs) by Histiocyte Society (HS) members or colleagues. In particular, for each case of LCH-AM, the database reports all the available data about clinical and biologic characteristics of the two tumors, as well about treatment and status at follow-up. The AM were categorized as: i) leukemias [acute lymphoblastic or myeloid leukemia (ALL and AML, respectively), other leukemias] and myeloproliferative disorders; ii) lymphomas [Hodgkin lymphoma (HL) and non-Hodgkin lymphomas (NHL)] and iii) solid tumors.

A total of 270 LCH-AM cases were documented, of which 116 (43%) occurred among children. After stratification by age at LCH diagnosis, using 18 years as cut-off between children and adults, we here provide details on the clinical characteristics in terms of LCH system involvement and affected organs, as well on the temporal relationship between the LCH and AM diagnoses, including details on the AM malignancy types. In 19 cases the LCH and the corresponding AM occurred in a different age group.

The data set is available for future studies in view of new insights of the genetic or environmental determinants of LCH and/or of treatment related subsequent neoplasms.

**Specifications Table**

**Table:** 

Subject	Health and medical sciences
Specific subject area	Hematology/Oncology – genetic predisposition and treatment related subsequent neoplasms
Type of data	Table
How the data were acquired	The LCH-AM data set was initiated in 1991. Firstly, a request to members of the Histiocyte Society (HS) was made to report any patient of any age they were currently treating, or had previously treated with LCH and another malignancy which may have occurred before, concurrently or after the LCH diagnosis. Further updates were made by reviewing abstracts at HS or other international meetings. Secondly, the scientific literature was periodically searched through the PubMed data base. For each case of the reported association, an ad *hoc* case report form (CRF) (attached as supplementary data) was requested to be completed by HS members, otherwise data were directly abstracted from the published manuscripts by the personnel involved in this study (Figure 1 and 2) . Data collection ended on June 2015.
Data format	Raw Analyzed
Description of data collection	LCH-AM cases reported in this article are mostly derived from previously published manuscripts which are listed (Table 1) in the data sources location section of this document, or from CRFs by HS members or colleagues. Non eligible cases (i.e. histiocytic disorder other than LCH, AM being a benign tumor) were excluded. Cases were already pseudo anonymized or completely anonymized at the moment of their reporting in the literature and are thus identifiable only through their study identification code.
Data source location	All the information available and collected through CRFs was stored in an ad hoc Access database in a secure institutional server at the IRCCS Istituto Giannina Gaslini, in Genoa, Italy. List of the primary data sources is reported in Table 1
Data accessibility	The full secondary dataset linked to the tables is publicly available via the Mendeley Data repository. Repository name: Mendeley Data Direct URL to data: https://data.mendeley.com/datasets/yvtfcr52x6/2
Related research article	Francesca Bagnasco, Stefanie Yvonne Zimmermann, Rudolph Maarten Egeler, Vasanta Rao Nanduri, Bruna Cammarata, Jean Donadieu, Thomas Lehrnbecher, Riccardo Haupt, Langerhans cell histiocytosis and associated malignancies: A retrospective analysis of 270 patients, European Journal of Cancer, Volume 172, 2022, Pages 138-145, ISSN 0959-8049, https://doi.org/10.1016/j.ejca.2022.03.036.

**Value of the Data**

• This dataset is the largest series to date of the association of 270 LCH cases with another malignancy and provides the relevant bibliography on published cases of LCH-AM.
• For each case of the LCH-AM association, the dataset provides details on the LCH characterization (system and organ involvement), pathology reports of LCH and AM (where available), treatment exposures and clinical course both of LCH and AM.
• This data can be used to generate hypotheses to investigate possible common pathways between the two malignancies which might then addressed by prospective data collection of clinical and biological data.
• Any researcher interested in the patho-physiology of LCH may use these data to identify unusual associations even with other rare tumors which might be interesting to analyze in the light of new genetic findings.

## 1 Data Description

The raw data described in this article are available in Mendeley Data, DOI: https://data.mendeley.com/datasets/yvtfcr52x6/2 [1]. Details on 270 LCH cases with another associated malignancy (AM) are shown in “LCH_Malignancy_raw_data.xlsx”. Detailed explanation of each column in the file, is reported in “*LCH_Malignancy_Variables_list.pdf*”. For each case, the reported data are on sex and ethnicity (classification according [2]), age at LCH diagnosis, extension of the disease, system(s) involved, histopathology, surgery, treatment including details on site and dose of any radiotherapy, list of chemotherapy or immunotherapy drugs used. Further information refer to the AM and in details: age at diagnosis, type of malignancy, stage and site of the primary and if the malignancy occurred within the previous radiation port used to treat the LCH, if any; type of treatment(s) (surgery, chemotherapy and radiotherapy) with details for the site and dose of radiotherapy. Other information, if available, referred to the vital status at follow-up, cause of death for deceased subjects and the clinical status (active or remission) of both the LCH and the AM.

**Fig. 1:**
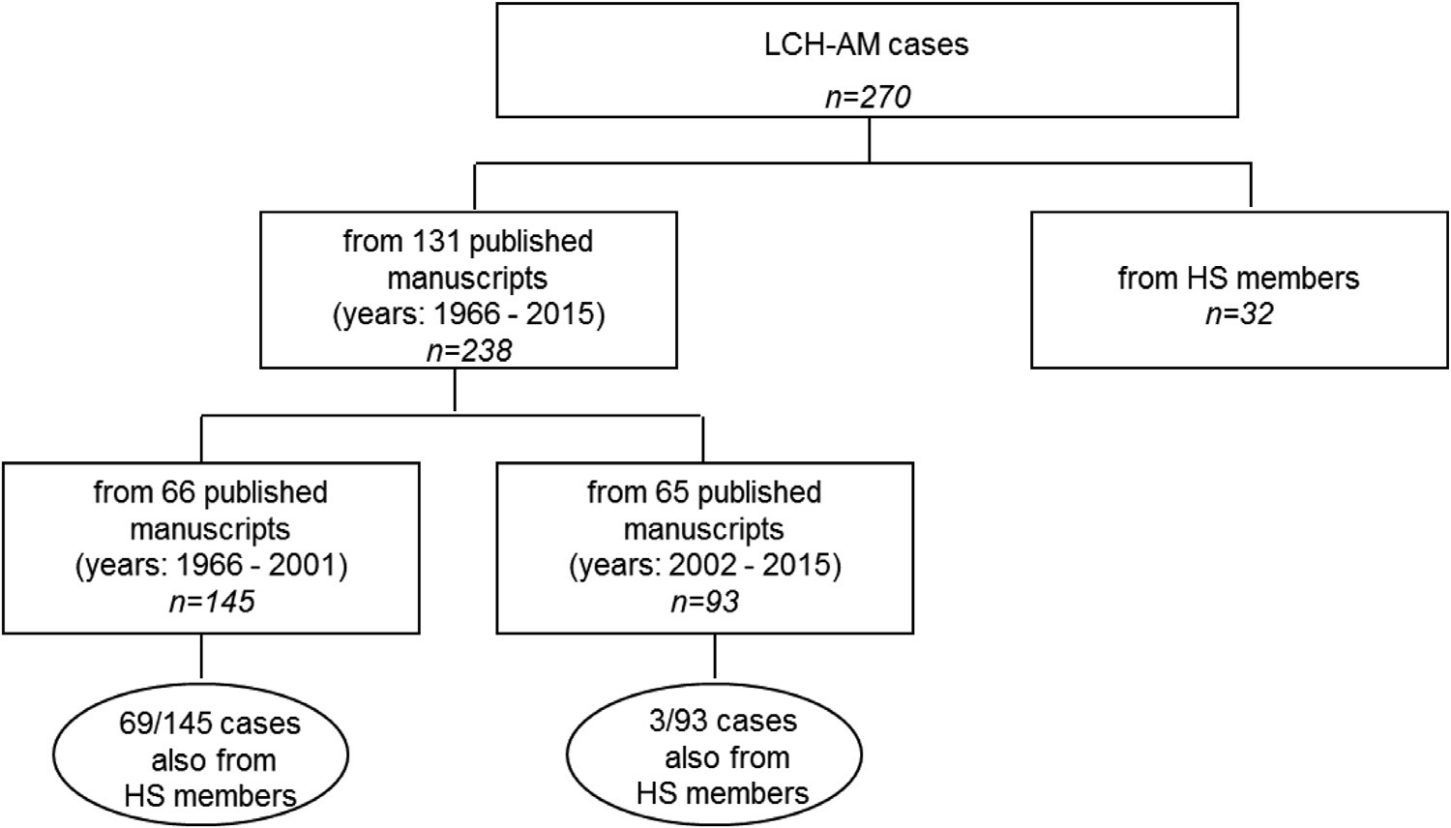
Data source of the 270 cases of LCH with associated malignancy-AM.

**Fig. 2:**
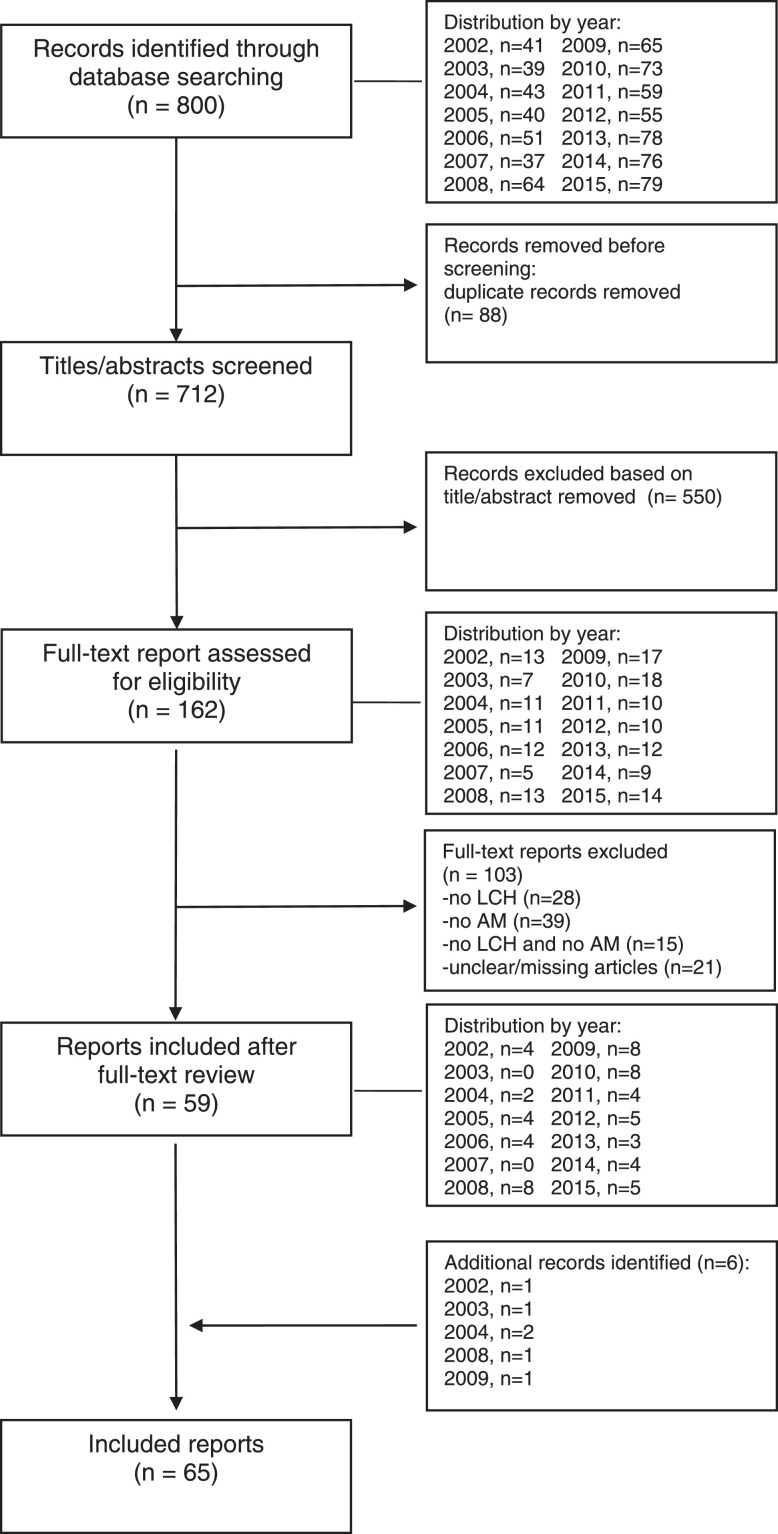
Flow diagram for selection of reports to be included in the LCH-AM dataset.

The study CRF is provided as a supplementary file. The data sources of the 270 cases are described in Fig. 1 and in Table 1 the primary data sources (all published manuscripts) are listed. Fig. 2 describes the periodic annual searches, from 2002 to 2015, through the PubMed data base. Table 2 reports details of the 270 cases in terms of system involvement (single system – SS, *vs*. multi system - MS), and affected organs. In the pediatric group (n=116), the skeleton was the most frequently affected system (both in SS- and MS-LCH; n=86; 74.1%), followed by skin (n=51; 44%), lymph nodes (n=26; 22.4%), liver and lungs (n=21 each, 18.1%). Among adults (n=154), the lungs (n=65; 42.2%) were the most affected organ followed by lymph nodes (n=47, 30.5%) and skin (n=35; 22.7%).

**Table 1:** Langerhans Cell Histiocytosis and other malignanicies - List of the primary data sources.

*Primary data sources*
1. Adu-Poku K, Thomas DW, Khan MK, Holgate CS, Smith ME. Langerhans cell histiocytosis in sequential discordant lymphoma. J Clin Pathol. 2005 Jan;58(1):104-6. doi: 10.1136/jcp.2003.015537
2. Al-Anazi KA, Alshehri A, Al-Zahrani HA, Al-Mohareb FI, Maghfoor I, Ajarim D. Successful outcome of Langerhans cell histiocytosis complicated by therapy-related myelodysplasia and acute myeloid leukemia: a case report. Cases J. 2008 Aug 18;1(1):101. doi: 10.1186/1757-1626-1-101.
3. Almanaseer IY, Kosova L, Pellettiere EV. Composite lymphoma with immunoblastic features and Langerhans' cell granulomatosis (histiocytosis X). Am J Clin Pathol. 1986 Jan;85(1):111-4. doi: 10.1093/ajcp/85.1.111
4. Aricò M, Comelli A, Bossi G, Raiteri E, Piombo M, Egeler RM. Langerhans cell histiocytosis and acute leukemia: unusual association in two cases. Med Pediatr Oncol. 1993;21(4):271-3. doi: 10.1002/mpo.2950210407.
5. Arrinda JM, Vilanova JR, Zabalza IE, Ortega FJ, Bilbao FJ, Rivera-Pomar JM. Solitary Langerhans' cell granulomatosis of the stomach associated with gastric carcinoma. Virchows Arch A Pathol Anat Histopathol. 1985;408(2-3):323-8. doi: 10.1007/BF00707995.
6. Aslan V, Akay OM, Durak B, Kabukcuoglu S, Gulbas Z. Langerhans cell histiocytosis with transformation to acute leukemia showing 45,X, t(8; 21), 5q-, -Y karyotype. Leuk Lymphoma. 2002 Aug;43(8):1683-5. doi: 10.1080/1042819021000003036.
7. Baikian B, Descamps V, Grossin M, Marinho E, Picard C, Aitken G, Sigal M, Crickx B, Belaich S. Histiocytose langerhansienne et leucémie de la lignée myélomonocytaire: une association non fortuite [Langerhans cell histiocytosis and myelomonocytic leukemia: a non-fortuitous association]. Ann Dermatol Venereol. 1999 May;126(5):409-11. French.
8. Basset F, Soler P, Wyllie L, Abelanet R, Le Charpentier M, Kreis B, Breathnach AS. Langerhans cells in a bronchiolar-alveolar tumour of lung. Virchows Arch A Pathol Anat Histol. 1974;362(4):315-30. doi: 10.1007/BF00427080.
9. Billings SD, Hans CP, Schapiro BL, Martin RW 3rd, Fivenson D, Fruland JE, Moores WB, Cotton J. Langerhans cell histiocytosis associated with myelodysplastic syndrome in adults. J Cutan Pathol. 2006 Feb;33(2):171-4. doi: 10.1111/j.0303-6987.2006.00299.x.
10. Burns BF, Colby TV, Dorfman RF. Langerhans' cell granulomatosis (histiocytosis X) associated with malignant lymphomas. Am J Surg Pathol. 1983 Sep;7(6):529-33. doi: 10.1097/00000478-198309000-00003.
11. Cavazza A, Pasquinelli G, Carlinfante G, Cenini E, Bonvicini U, Gardini G. Nodular Langerhans cell histiocytosis of the liver in an adult with colonic adenocarcinoma. Histopathology. 1999 Mar;34(3):273-5.
12. Chang CC, Somach SC. Firm erythematous papules of scalp in a woman with a history of breast cancer. Arch Pathol Lab Med. 2001 Oct;125(10):1379-80. doi: 10.5858/2001-125-1379-PQC.
13. Chiles LR, Christian MM, McCoy DK, Hawkins HK, Yen AH, Raimer SS. Langerhans cell histiocytosis in a child while in remission for acute lymphocytic leukemia. J Am Acad Dermatol. 2001 Dec;45(6 Suppl):S233-4. doi: 10.1067/mjd.2001.104964.
14. Churn M, Davies C, Slater A. Synchronous bilateral carcinoma of the breasts occurring in a young woman with a history of Langerhans' cell histiocytosis in infancy. Clin Oncol (R Coll Radiol). 1999;11(6):410-3. doi: 10.1053/clon.1999.9094.
15. Claudy AL, Larbre B, Colomb M, Levigne V, Deville V. Letterer-Siwe disease and subacute monocytic leukemia. J Am Acad Dermatol. 1989 Nov;21(5 Pt 2):1105-6. doi: 10.1016/s0190-9622(89)70306-6.
16. Coli A, Bigotti G, Ferrone S. Histiocytosis X arising in Hodgkin's disease: immunophenotypic characterization with a panel of monoclonal antibodies. Virchows Arch A Pathol Anat Histopathol. 1991;418(4):369-73. doi: 10.1007/BF01600168.
17. Das DK, Sheikh ZA, Alansary TA, Amir T, Al-Rabiy FN, Junaid TA. A case of Langerhans' cell histiocytosis associated with Hodgkin's lymphoma: Fine-needle aspiration cytologic and histopathological features. Diagn Cytopathol. 2016 Feb;44(2):128-32. doi: 10.1002/dc.23392.
18. de Camargo B, Alves AC, Gorender EF, Bianchi A. Association of malignancy and Langerhans' cell histiocytosis: report of three cases. Med Pediatr Oncol. 1993;21(6):451-3. doi: 10.1002/mpo.2950210612.
19. de Pinieux G, Dulmet E, Chapelier A, Beuzeboc P, Debré B. Tumeur germinale non séminomateuse du testicule et histiocytose langerhansienne pulmonaire: une association fortuite? [Non-seminomatous germ cell tumor of the testis and pulmonary Langerhans-cell histiocytosis: a fortuitous association?]. Presse Med. 1997 Feb 8;26(3):118-9. French.
20. Dufour C, Lanciotti M, Micalizzi C, Valetto A, Haupt R. Non-identical twin sisters concordant for Langerhans cell histiocytosis and discordant for secondary acute promyelocytic leukemia. Med Pediatr Oncol. 2001 Jul;37(1):70-2. doi: 10.1002/mpo.1169.
21. Edelbroek JR, Vermeer MH, Jansen PM, Stoof TJ, van der Linden MM, Horváth B, van Baarlen J, Willemze R. Langerhans cell histiocytosis first presenting in the skin in adults: frequent association with a second haematological malignancy. Br J Dermatol. 2012 Dec;167(6):1287-94. doi: 10.1111/j.1365-2133.2012.11169.x.
22. Egeler RM, Neglia JP, Aricò M, Favara BE, Heitger A, Nesbit ME, Nicholson HS. The relation of Langerhans cell histiocytosis to acute leukemia, lymphomas, and other solid tumors. The LCH-Malignancy Study Group of the Histiocyte Society. Hematol Oncol Clin North Am. 1998 Apr;12(2):369-78. doi: 10.1016/s0889-8588(05)70516-5.
23. Feldman AL, Berthold F, Arceci RJ, Abramowsky C, Shehata BM, Mann KP, Lauer SJ, Pritchard J, Raffeld M, Jaffe ES. Clonal relationship between precursor T-lymphoblastic leukaemia/lymphoma and Langerhans-cell histiocytosis. Lancet Oncol. 2005 Jun;6(6):435-7. doi: 10.1016/S1470-2045(05)70211-4.
24. Ferrari A, Fabietti P, Vessecchia G, Laffranchi A, Lombardi L, Massimino M, Fossati-Bellani F, Giardini R. Langerhans cell histiocytosis arising after Hodgkin's disease. Pediatr Hematol Oncol. 1997 Nov-Dec;14(6):585-8. doi: 10.3109/08880019709030916.
25. Feuillet S, Louis L, Bergeron A, Berezne A, Dubreuil ML, Polivka M, Oksenhendler E, Tazi A. Pulmonary Langerhans cell histiocytosis associated with Hodgkin's lymphoma. Eur Respir Rev. 2010 Mar;19(115):86-8. doi: 10.1183/09059180.00007509.
26. Fischer A, Jones L, Lowis SP. Concurrent Langerhans cell histiocytosis and neuroblastoma. Med Pediatr Oncol. 1999 Mar;32(3):223-4. doi: 10.1002/(sici)1096-911x(199903)32:3<223::aid-mpo11>3.0.co;2-s
27. Fontana J, Koss W, McDaniel D, Jenkins J 3rd, Welton W. Histiocytosis X and acute monocytic leukemia. Biologic illustration of the monocyte phagocytic system. Am J Med. 1987 Jan;82(1):137-42. doi: 10.1016/0002-9343(87)90391-3.
28. Foucar K, Foucar E, Weiler R, Stuart T. Correspondence re: R.G. Lee, R.M. Braziel, and P. Stenzel. Gastrointestinal involvement in Langerhans cell histiocytosis (histiocytosis X): diagnosis by rectal biopsy. Mod Pathol 3:154, 1990. Mod Pathol. 1991 Mar;4(2):284.
29. Foulet-Rogé A, Josselin N, Guyetant S, Gardet JJ, Besancon A, Saint-André JP, Fabiani B. Incidental langerhans cell histiocytosis of thyroid: case report and review of the literature. Endocr Pathol. 2002 Fall;13(3):227-33. doi: 10.1385/ep:13:3:227.
30. Ghosn MG, Haddad AC, Nassar MN, Abadjian GA, El Karak FR, Aftimos PG. Acute myeloid leukemia and Langerhans' cell histiocytosis: multiple theories for an unusual presentation. Leuk Res. 2010 Mar;34(3):406-8. doi: 10.1016/j.leukres.2009.07.025. Epub 2009 Aug 11.
31. Gimeno E, Cervera M, Trampal C, Solano A, Villalba G, Serrano S, Besses C, Salar A. Langerhans' cell histiocytosis mimicking relapse in a patient with follicular lymphoma. Ann Hematol. 2008 Aug;87(8):675-6. doi: 10.1007/s00277-008-0459-y. Epub 2008 Mar 20.
32. Gold DG, Neglia JP, Potish RA, Dusenbery KE. Second neoplasms following megavoltage radiation for pediatric tumors. Cancer. 2004 Jan 1;100(1):212-3. doi: 10.1002/cncr.11870
33. Greaves WO, Bueso-Ramos C, Fayad L. Classical Hodgkin's lymphoma associated with Langerhans cell histiocytosis: multiagent chemotherapy resulted in histologic resolution of both the classical Hodgkin's lymphoma and Langerhans cell proliferation components. J Clin Oncol. 2011 Feb 1;29(4):e76-8. doi: 10.1200/JCO.2010.31.2413.
34. Hale GA, Greenwood MF, Geil JD, Moscow JA. Langerhans cell histiocytosis after therapy for a malignant germ cell tumor of the central nervous system. J Pediatr Hematol Oncol. 2000 Jul-Aug;22(4):355-7. doi: 10.1097/00043426-200007000-00015.
35. Hammami H, Zaraa I, El Euch D, Chelly I, Haouet S, Mokni M, Ben Osman A. Letterer-Siwe disease associated with chronic myelomyonocytic leukemia: a fortuitous association? Acta Dermatovenerol Alp Pannonica Adriat. 2010;19(1):45-8.
36. Haupt R, Comelli A, Rosanda C, Sessarego M, De Bernardi B. Acute myeloid leukemia after single-agent treatment with etoposide for Langerhans' cell histiocytosis of bone. Am J Pediatr Hematol Oncol. 1993 May;15(2):255-7. doi: 10.1097/00043426-199305000-00015.
37. Haupt R, Fears TR, Heise A, Gadner H, Loiacono G, De Terlizzi M, Tucker MA. Risk of secondary leukemia after treatment with etoposide (VP-16) for Langerhans' cell histiocytosis in Italian and Austrian-German populations. Int J Cancer. 1997 Mar 28;71(1):9-13. doi: 10.1002/(sici)1097-0215(19970328)71:1<9::aid-ijc3>3.0.co;2-y.
38. Haupt R, Fears TR, Rosso P, Colella R, Loiacono G, de Terlizzi M, Mancini A, Comelli A, Indolfi P, Donfrancesco A, et al. Increased risk of secondary leukemia after single-agent treatment with etoposide for Langerhans' cell histiocytosis. Pediatr Hematol Oncol. 1994 Sep-Oct;11(5):499-507. doi: 10.3109/08880019409141688.
39. Hirsh R, Giri D, Griffith R, Stone R, Ready N. Langerhans cell histiocytosis following acute leukemia in an adult. Am J Hematol. 2009 Oct;84(10):693-4. doi: 10.1002/ajh.21490.
40. Horibe K, Matsushita T, Numata S, Miyajima Y, Katayama I, Kitabayashi T, Yanai M, Sekiguchi N, Egi S. Acute promyelocytic leukemia with t(15;17) abnormality after chemotherapy containing etoposide for Langerhans cell histiocytosis. Cancer. 1993 Dec 15;72(12):3723-6. doi: 10.1002/1097-0142(19931215)72:12<3723::aid-cncr2820721226>3.0.co;2-y.
41. Hwang YY, Tsui P, Leung RY, Kwong YL. Disseminated Langerhans cell histiocytosis associated with acute myeloid leukaemia: complete remission with daunorubicin and cytarabine. Ann Hematol. 2013 Jan;92(2):267-8. doi: 10.1007/s00277-012-1555-6.
42. Ibarrola de Andrés C, Toscano R, Lahuerta JJ, Martínez-González MA. Simultaneous occurrence of Hodgkin's disease, nodal Langerhans' cell histiocytosis and multiple myeloma IgA(kappa). Virchows Arch. 1999 Mar;434(3):259-62. doi: 10.1007/s004280050338.
43. Iwasaki T, Takahashi I, Nagashima T, Igawa S, Komatsu S, Honma M, Ishida-Yamamoto A, Iizuka H. Cutaneous Langerhans cell histiocytosis in elderly with chronic myelomonocytic leukemia. J Dermatol. 2014 Mar;41(3):262-5. doi: 10.1111/1346-8138.12417.
44. Izikson L, Vanderpool J, Brodsky G, Mihm MC Jr, Zembowicz A. Combined basal cell carcinoma and Langerhans cell histiocytosis of the scrotum in a patient with occupational exposure to coal tar and dust. Int J Dermatol. 2004 Sep;43(9):678-80. doi: 10.1111/j.1365-4632.2004.02178.x.
45. Jamaati HR, Shadmehr MB, Saidi B, Khosravi A, Arab M, Mohammadi F. Langerhans cell histiocytosis of the lung and thyroid, co-existing with papillary thyroid cancer. Endocr Pathol. 2009 Summer;20(2):133-6. doi: 10.1007/s12022-009-9068-0.
46. Jeong TD, Jang S, Park CJ, Chi HS, Lee JH. A case of Langerhans cell histiocytosis following acute basophilic leukemia. Ann Hematol. 2013 Jan;92(1):137-9. doi: 10.1007/s00277-012-1542-y.
47. Kager L, Heise A, Minkov M, Möbius D, Kotte W, Schulte-Overberg U, Henze G, Gadner H. Occurrence of acute nonlymphoblastic leukemia in two girls after treatment of recurrent, disseminated Langerhans cell histiocytosis. Pediatr Hematol Oncol. 1999 May-Jun;16(3):251-6. doi: 10.1080/088800199277317.
48. Kaiserling E, Horny HP. Dermal Langerhans' cell tumor in chronic myelomonocytic leukemia. Ultrastruct Pathol. 1988 Mar-Apr;12(2):209-19. doi: 10.3109/01913128809058219
49. Karadeniz C, Sarialioğlu F, Göğüş S, Akyüz C, Küçükali T, Kutluk T, Büyükpamukçu M. Multiple primary malignancy: a report on Langerhans cell histiocytosis associated with Hodgkin's disease. Turk J Pediatr. 1991 Jul-Sep;33(3):185-90.
50. Keen CE, Philip G, Parker BC, Souhami RL. Unusual bony lesions of histiocytosis X in a patient previously treated for Hodgkin's disease. Pathol Res Pract. 1990 Aug;186(4):519-25; discussion 526. doi: 10.1016/S0344-0338(11)80475-9.
51. Kjeldsberg CR, Kim H. Eosinophilic granuloma as an incidental finding in malignant lymphoma. Arch Pathol Lab Med. 1980 Mar;104(3):137-40.
52. Ko YH, Kim WS, Kim Y. Expression of CD56 antigen in Langerhans cell histiocytosis associated with T-lymphoblastic lymphoma in a same lymph node. Virchows Arch. 2006 Jan;448(1):90-4. doi: 10.1007/s00428-005-0093-1. Epub 2005 Oct 14.
53. Koral K, Roy D, Timmons CF, Gargan L, Bowers DC. Low-grade bone lesions in survivors of childhood medulloblastoma/primitive neuroectodermal tumor. Acad Radiol. 2012 Jan;19(1):35-9. doi: 10.1016/j.acra.2011.08.018
54. Lee DA, Tatevian N, Herring RA, McClain KL. EBV+ lymphoproliferative disease following prolonged chemotherapy for refractory LCH. Pediatr Blood Cancer. 2008 Mar;50(3):728-30. doi: 10.1002/pbc.21121.
55. Lee JS, Ko GH, Kim HC, Jang IS, Jeon KN, Lee JH. Langerhans cell sarcoma arising from Langerhans cell histiocytosis: a case report. J Korean Med Sci. 2006 Jun;21(3):577-80. doi: 10.3346/jkms.2006.21.3.577.
56. L'Hoste RJ Jr, Arrowsmith WR, Leonard GL, McGaw H. Eosinophilic granuloma occurring in a patient with Hodgkin disease. Hum Pathol. 1982 Jun;13(6):592-5. doi: 10.1016/s0046-8177(82)80278-5.
57. Li S, Borowitz MJ. CD79a(+) T-cell lymphoblastic lymphoma with coexisting Langerhans cell histiocytosis. Arch Pathol Lab Med. 2001 Jul;125(7):958-60. doi: 10.5858/2001-125-0958-CTCLLW.
58. Licci S, Boscaino A, De Palma M, Del Nonno F, D'Antonio A. Concurrence of marginal zone B-cell lymphoma MALT-type and Langerhans cell histiocytosis in a thyroid gland with Hashimoto disease. Ann Hematol. 2008 Oct;87(10):855-7. doi: 10.1007/s00277-008-0489-5.
59. Lindley R, Hoile R, Schofield J, Ashton-Key M. Langerhans cell histiocytosis associated with papillary carcinoma of the thyroid. Histopathology. 1998 Feb;32(2):180. doi: 10.1046/j.1365-2559.1998.00285.x.
60. Llamas-Velasco M, Cannata J, Dominguez I, García-Noblejas A, Aragües M, Fraga J, Arranz R. Coexistence of Langerhans cell histiocytosis, Rosai-Dorfman disease and splenic lymphoma with fatal outcome after rapid development of histiocytic sarcoma of the liver. J Cutan Pathol. 2012 Dec;39(12):1125-30. doi: 10.1111/cup.12013
61. Lombard CM, Medeiros LJ, Colby TV. Pulmonary histiocytosis X and carcinoma. Arch Pathol Lab Med. 1987 Apr;111(4):339-41.
62. Lopes LF, de Camargo B. Secondary acute promyelocytic leukemia after treatment with etoposide for Langerhans cell histiocytosis (LCH). Med Pediatr Oncol. 1999 Apr;32(4):315. doi: 10.1002/(sici)1096-911x(199904)32:4<315::aid-mpo17>3.0.co;2-6.
63. Lovrenski A, Djurić M, Klemt I, Eri Z, Panjković M, Tegeltija D, Povazan D. Multisystem Langerhans cell histiocytosis coexisting with metastasizing adenocarcinoma of the lung: a case report. Vojnosanit Pregl. 2013 Dec;70(12):1159-61. doi: 10.2298/vsp1312159l.
64. Mahoney DH Jr, McClain KL, Hanson IC, Taylor LD, Steuber CP. Acquired immune deficiency, myelodysplasia, and acute nonlymphocytic leukemia associated with monosomy 7 and t(3;3) (q21;q26) in a child with Langerhans cell histiocytosis. Am J Pediatr Hematol Oncol. 1989 Summer;11(2):153-7.
65. Marzano AV, Gasparini G, Grammatica A, de Juli E, Caputo R. Langerhans cell histiocytosis and thyroid carcinoma. Br J Dermatol. 1998 May;138(5):909-10. doi: 10.1046/j.1365-2133.1998.02239.x.
66. Matsuzaki A, Inamitsu T, Watanabe T, Ohga S, Ishii E, Nagotoshi Y, Tasaka H, Suda M, Ueda K. Acute promyelocytic leukaemia in a patient treated with etoposide for Langerhans cell histiocytosis. Br J Haematol. 1994 Apr;86(4):887-9. doi: 10.1111/j.1365-2141.1994.tb04851.x.
67. Melexopoulou CA, Sgouros J, Argyriou P, Tsitsimelis D, Aravantinos G, Samantas E. Pulmonary Langerhans cell histiocytosis in a patient previously treated for germ cell tumor. J BUON. 2010 Jan-Mar;15(1):194-5.
68. Michetti G, Cottini M, Scelsi L, Pugliese C, Minio A, Arnone P, Bamberga M, Ori Belometti M, Scelsi R. Langerhans' cell granulomatosis and Hodgkin's lymphoma. Report of a case. Minerva Med. 1996 May;87(5):243-7.
69. Miller DR. Raised foetal haemoglobin in childhood leukaemia. Br J Haematol. 1969 Jul;17(1):103-12. doi: 10.1111/j.1365-2141.1969.tb05668.x.
70. Misaki H, Yamauchi T, Arai H, Yamamoto S, Sutoh H, Yoshida A, Tsutani H, Eguchi M, Nagoshi H, Naiki H, Baba H, Ueda T, Yamakawa M. Secondary malignant fibrous histiocytoma following refractory langerhans cell histiocytosis. J Clin Exp Hematop. 2009 May;49(1):33-7. doi: 10.3960/jslrt.49.33.
71. Möricke A, Reiter A, Zimmermann M, Gadner H, Stanulla M, Dördelmann M, Löning L, Beier R, Ludwig WD, Ratei R, Harbott J, Boos J, Mann G, Niggli F, Feldges A, Henze G, Welte K, Beck JD, Klingebiel T, Niemeyer C, Zintl F, Bode U, Urban C, Wehinger H, Niethammer D, Riehm H, Schrappe M; German-Austrian-Swiss ALL-BFM Study Group. Risk-adjusted therapy of acute lymphoblastic leukemia can decrease treatment burden and improve survival: treatment results of 2169 unselected pediatric and adolescent patients enrolled in the trial ALL-BFM 95. Blood. 2008 May 1;111(9):4477-89. doi: 10.1182/blood-2007-09-112920. Epub 2008 Feb 19. Erratum in: Blood. 2009 Apr 30;113(18):4478. Dosage error in article text.
72. Moschovi M, Adamaki M, Vlahopoulos S, Rodriguez-Galindo C. Synchronous and metachronous thyroid cancer in relation to Langerhans cell histiocytosis; involvement of V600E BRAF-mutation? Pediatr Blood Cancer. 2015 Jan;62(1):173-4. doi: 10.1002/pbc.25173.
73. Murray PA, Hall PA. Histiocytosis X mimicking recurrent malignant disease: a report of two cases. Clin Radiol. 1988 May;39(3):310-2. doi: 10.1016/s0009-9260(88)80550-6.
74. Naresh KN, Zokaie S, Madhavan KS, Chu A. The Hammersmith Hospital hematopathology case of the month: myeloid sarcoma with Langerhans cell proliferation. Leuk Lymphoma. 2010 Jun;51(6):1128-34. doi: 10.3109/10428191003777989. Erratum in: Leuk Lymphoma. 2010 Aug;51(8):1582. Chzu, Anthony [corrected to Chu, Anthony].
75. Narui R, Yagasaki H, Takahashi Y, Hama A, Nishio N, Muramatsu H, Shimoyama Y, Kojima S. Concurrent Langerhans cell histiocytosis and nephroblastoma. Pediatr Blood Cancer. 2009 May;52(5):662-4. doi: 10.1002/pbc.21927.
76. Neumann MP, Frizzera G. The coexistence of Langerhans' cell granulomatosis and malignant lymphoma may take different forms: report of seven cases with a review of the literature. Hum Pathol. 1986 Oct;17(10):1060-5. doi: 10.1016/s0046-8177(86)80091-0.
77. Ng WK, Lam KY, Ng IO. Langerhans' cell histiocytosis: possible association with malignant germ cell tumour. J Clin Pathol. 1995 Oct;48(10):963-5. doi: 10.1136/jcp.48.10.963
78. Niksarlioglu YO, Gurel B, Tezel GG, Firat P, Dogan R, Kars A, Coplu L. Langerhans' cell histiocytosis mimicking metastatic carcinoma of the lung. Respirology. 2009 Apr;14(3):456-8. doi: 10.1111/j.1440-1843.2008.01474.x. Epub 2009 Jan 27.
79. Numakura S, Morikawa T, Ushiku T, Toyoshima T, Fukayama M. Langerhans cell histiocytosis of the urinary bladder in a patient with bladder cancer previously treated with intravesical Bacillus Calmette-Guérin therapy. Pathol Res Pract. 2014 Feb;210(2):123-6. doi: 10.1016/j.prp.2013.11.005.
80. Ohtsuki Y, Uomoto M, Hachisuka Y, Kato M, Iguchi M, Lee GH, Furihata M. A rare case of coexistence of pulmonary adenocarcinoma with Langerhans' cell histiocytosis. Med Mol Morphol. 2008 Sep;41(3):175-8. doi: 10.1007/s00795-008-0402-2.
81. O'Kane D, Jenkinson H, Carson J. Langerhans cell histiocytosis associated with breast carcinoma successfully treated with topical imiquimod. Clin Exp Dermatol. 2009 Dec;34(8):e829-32. doi: 10.1111/j.1365-2230.2009.03569.x.
82. Pan Z, Bland KI, Wei S. Composite cutaneous atypical vascular lesion and Langerhans cell histiocytosis after radiation for breast carcinoma: can radiation induce Langerhans cell histiocytosis? Dermatol Online J. 2011 Dec 15;17(12):6.
83. Park IS, Park IK, Kim EK, Kim S, Jeon SR, Huh JR, Suh CW. Langerhans cell histiocytosis followed by Hodgkin's lymphoma. Korean J Intern Med. 2012 Dec;27(4):459-62. doi: 10.3904/kjim.2012.27.4.459.
84. Pastor-Jané L, Escoda-Teigell L, Martínez-González S, Turégano-Fuentes P, Requena-Caballero L. Multiorgan histiocytosis after B-cell acute lymphoblastic leukemia. Am J Dermatopathol. 2011 Jul;33(5):516-20. doi: 10.1097/DAD.0b013e3181ed3a12.
85. Patel P, Talpur R, Duvic M. Langerhans cell histiocytosis arising from a BCC: a case report and review of the literature. Cutis. 2010 Jun;85(6):295-300.
86. Pimentel A, Haupt R, Sihelnik SA, Kimmel WB, Swierczynski SL. Focal Langerhans cell histiocytosis (LCH) coexisting with renal cell carcinoma. J Clin Oncol. 2011 Feb 10;29(5):e107-9. doi: 10.1200/JCO.2010.30.9344.
87. Pistamaltzian N, Nikolaidi A, Raftogiannis M, Economou A, Mourtzoukos S and Athanasiadis I. Langerhans cell histiocytosis following treatment for testicular cancer. A case report and literature review. Forum of Clinical Oncology, vol.6, no.1, 2015, pp.6-9. https://doi.org/10.1515/fco-2015-0002
88. Quintanilla-Martinez L, Zukerberg LR, Harris NL. Prethymic adult lymphoblastic lymphoma. A clinicopathologic and immunohistochemical analysis. Am J Surg Pathol. 1992 Nov;16(11):1075-84. doi: 10.1097/00000478-199211000-00006.
89. Raj A, Bendon R, Moriarty T, Suarez C, Bertolone S. Langerhans cell histiocytosis following childhood acute lymphoblastic leukemia. Am J Hematol. 2001 Dec;68(4):284-6. doi: 10.1002/ajh.10004.
90. Rayburg M, Towbin A, Yin H, Maugans T, Maurer B, Nagarajan R, Weiss B. Langerhans cell histiocytosis in a patient with stage 4 neuroblastoma receiving oral fenretinide. Pediatr Blood Cancer. 2009 Dec;53(6):1111-3. doi: 10.1002/pbc.22200.
91. Régis A, Ben Salem D, Lambert A, Couaillier JF, Casasnovas O, Piard F, Krausé D. Association rare de l'histiocytose Langerhansienne pulmonaire et d'un lymphome malin: à propos de deux cas [Concomitant pulmonary Langerhans cell histiocytosis and malignant lymphoma: report of two cases]. J Radiol. 2009 Jan;90(1 Pt 1):66-8. French. doi: 10.1016/s0221-0363(09)70081-2.
92. Rodig SJ, Payne EG, Degar BA, Rollins B, Feldman AL, Jaffe ES, Androkites A, Silverman LB, Longtine JA, Kutok JL, Fleming MD, Aster JC. Aggressive Langerhans cell histiocytosis following T-ALL: clonally related neoplasms with persistent expression of constitutively active NOTCH1. Am J Hematol. 2008 Feb;83(2):116-21. doi: 10.1002/ajh.21044.
93. Rodriguez-Galindo C, Jeng M, Khuu P, McCarville MB, Jeha S. Clofarabine in refractory Langerhans cell histiocytosis. Pediatr Blood Cancer. 2008 Nov;51(5):703-6. doi: 10.1002/pbc.21668.
94. Roufosse C, Lespagnard L, Salés F, Bron D, Dargent JL. Langerhans' cell histiocytosis associated with simultaneous lymphocyte predominance Hodgkin's disease and malignant melanoma. Hum Pathol. 1998 Feb;29(2):200-1. doi: 10.1016/s0046-8177(98)90236-2.
95. Sadoun D, Vaylet F, Valeyre D, Natali F, Georges R, Allard P, Battesti JP. Bronchogenic carcinoma in patients with pulmonary histiocytosis X. Chest. 1992 Jun;101(6):1610-3. doi: 10.1378/chest.101.6.1610.
96. Safali M, McCutcheon JM, Wright DH. Langerhans cell histiocytosis of lymph nodes: draining a papillary carcinoma of the thyroid. Histopathology. 1997 Jun;30(6):599-603. doi: 10.1046/j.1365-2559.1997.5590802.x.
97. Saiz A, Martinez MA, Grande C, Vanaclocha F. Langerhans' cell histiocytosis in an adult with acute myelogenous leukaemia. Virchows Arch. 2004 Jul;445(1):93-5. doi: 10.1007/s00428-004-1005-5.
98. Saiz E, Bakotic BW. Isolated Langerhans cell histiocytosis of the thyroid: a report of two cases with nuclear imaging-pathologic correlation. Ann Diagn Pathol. 2000 Feb;4(1):23-8. doi: 10.1016/s1092-9134(00)90006-6.
99. Sajjad SM, Luna MA. Primary pulmonary histiocytosis X in two patients with Hodgkin's disease. Thorax. 1982 Feb;37(2):110-3. doi: 10.1136/thx.37.2.110.
100. Schmitt-Graeff AH, Duerkop H, Vollmer-Kary B, Haxelmans S, Nitschke R, Fisch P, Germing U, Stein H. Clonal relationship between langerhans cell histiocytosis and myeloid sarcoma. Leukemia. 2012 Jul;26(7):1707-10. doi: 10.1038/leu.2012.27
101. Shamsian BS, Goudarzipour K, Alavi S, Jadali F, Gharib A, Aghakhani R, Arzanian MT, Rezaei N. Langerhans cell histiocytosis in a child with non-Hodgkin lymphoma. J Pediatr Hematol Oncol. 2010 Aug;32(6):e245-6. doi: 10.1097/MPH.0b013e3181e5e1c4.
102. Shanley DJ, Lerud KS, Luetkehans TJ. Development of pulmonary histiocytosis X after chemotherapy for Hodgkin disease. AJR Am J Roentgenol. 1990 Oct;155(4):741-2. doi: 10.2214/ajr.155.4.1698017.
103. Shiohama T, Ochiai H, Hishiki T, Yoshida H, Kohno Y. Coexistence of neuroblastoma detected on staging of Langerhans cell histiocytosis. Pediatr Int. 2014 Aug;56(4):608-10. doi: 10.1111/ped.12292.
104. Shiohama T, Ochiai H, Hishiki T, Yoshida H, Kohno Y. Coexistence of neuroblastoma detected on staging of Langerhans cell histiocytosis. Pediatr Int. 2014 Aug;56(4):608-10. doi: 10.1111/ped.12292.
105. Silva ML, Land MG, Maradei S, Otero L, Veith M, Brito G, Klumb C, Fernandez T, Pombo-de-Oliveira MS. Translocation (11;11)(p13- p15;q23) in a child with therapy-related acute myeloid leukemia following chemotherapy with DNA-topoisomerase II inhibitors for Langerhans cell histiocytosis. Cancer Genet Cytogenet. 2002 May;135(1):101-2. doi: 10.1016/s0165-4608(01)00638-0.
106. Simonart T, Urbain F, Verdebout JM, Feoli F, Lespagnard L, Dargent JL. Langerhans' cell histiocytosis arising at the site of basal cell carcinoma excision. J Cutan Pathol. 2000 Oct;27(9):476-8. doi: 10.1034/j.1600-0560.2000.027009476.x.
107. Simons M, Van De Nieuwenhof HP, Van Der Avoort IA, Bulten J, De Hullu JA. A patient with lichen sclerosus, Langerhans cell histiocytosis, and invasive squamous cell carcinoma of the vulva. Am J Obstet Gynecol. 2010 Aug;203(2):e7-10. doi: 10.1016/j.ajog.2010.04.023.
108. Soler N, Barberà JA, Ramirez J, Batllé M, Rozman C, Rodriguez-Roisin R. Pulmonary Langerhans' cell histiocytosis following autologous haemopoietic progenitor cell transplantation. Respir Med. 1998 Oct;92(10):1253-5. doi: 10.1016/s0954-6111(98)90430-9.
109. Surico G, Muggeo P, Rigillo N, Gadner H. Concurrent Langerhans cell histiocytosis and myelodysplasia in children. Med Pediatr Oncol. 2000 Oct;35(4):421-5. doi: 10.1002/1096-911x(20001001)35:4<421::aid-mpo5>3.0.co;2-h.
110. Tatsumi T, Shimazaki C, Araki SI, Sudo Y, Yamagata N, Ashihara E, Goto H, Inaba T, Fujita N, Nakagawa M, Misawa SI, Imashuku S. Philadelphia chromosome-positive acute lymphoblastic leukemia after therapy for Langerhans cell histiocytosis. Am J Hematol. 1997 Jan;54(1):88.
111. Taylor JL, Quiñones Maymí DM, Sporn TA, McAdam HP, Wahidi MM. Multiple lung nodules in a woman with a history of melanoma. Respiration. 2003 Sep-Oct;70(5):544-8. doi: 10.1159/000074217.
112. Texier L, Maleville J. Granulome éosinophile périorificiel et pulmonaire (histiocytosis X). Leucémie à monocytes terminale. (A propos de deux observations) [Periorificial and pulmonary eosinophilic granuloma (histiocytosis-X). Terminal monocytic leukemia. (Apropos of 2 cases)]. Bull Soc Fr Dermatol Syphiligr. 1966 Dec;73(6):935-43. French.
113. Thompson LD, Wenig BM, Adair CF, Smith BC, Heffess CS. Langerhans cell histiocytosis of the thyroid: a series of seven cases and a review of the literature. Mod Pathol. 1996 Feb;9(2):145-9.
114. Tirilomis T, Zenker D, Mirzaie M, Dalichau H. Pulmonary Langerhans' cell histiocytosis (histiocytosis X) following metastasizing malignant melanoma. Swiss Med Wkly. 2002 Jun 1;132(21-22):285-7.
115. Tomashefski JF, Khiyami A, Kleinerman J. Neoplasms associated with pulmonary eosinophilic granuloma. Arch Pathol Lab Med. 1991 May;115(5):499-506.
116. Trebo MM, Attarbaschi A, Mann G, Minkov M, Kornmüller R, Gadner H. Histiocytosis following T-acute lymphoblastic leukemia: a BFM study. Leuk Lymphoma. 2005 Dec;46(12):1735-41. doi: 10.1080/10428190500160017.
117. Tsuji T, Nakamura S, Tanaka M. Pulmonary Langerhans cell histiocytosis associated with lingual carcinoma. Intern Med. 2004 Aug;43(8):713-7. doi: 10.2169/internalmedicine.43.713.
118. Uskul BT, Turker H, Bayraktar OU, Onemli M. Bronchogenic carcinoma developing during a long-term course of pulmonary Langerhans' cell histiocytosis. Intern Med. 2009;48(5):359-62. doi: 10.2169/internalmedicine.48.1552. Epub 2009 Mar 2.
119. Valdivielso M, Bneno C. Extensive basal cell carcinoma and disseminated lesions in Hand-Schüller-Christian disease. Acta Derm Venereol. 2005;85(1):86-7. doi: 10.1080/00015550410022267.
120. Van Heerde P, Maarten Egeler R. The cytology of Langerhans cell histiocytosis (histiocytosis X). Cytopathology. 1991;2(3):149-58. doi: 10.1111/j.1365-2303.1991.tb00399.x.
121. Vassallo R, Ryu JH, Schroeder DR, Decker PA, Limper AH. Clinical outcomes of pulmonary Langerhans'-cell histiocytosis in adults. N Engl J Med. 2002 Feb 14;346(7):484-90. doi: 10.1056/NEJMoa012087.
122. Willis B, Ablin A, Weinberg V, Zoger S, Wara WM, Matthay KK. Disease course and late sequelae of Langerhans' cell histiocytosis: 25-year experience at the University of California, San Francisco. J Clin Oncol. 1996 Jul;14(7):2073-82. doi: 10.1200/JCO.1996.14.7.2073.
123. Wollina U, Kaatz M, Krönert C, Schönlebe J, Schmalenberg H, Schreiber G, Köstler E, Haroske G. Cutaneous Langerhans cell histiocytosis with subsequent development of haematological malignancies. Report of two cases. Acta Dermatovenerol Alp Pannonica Adriat. 2006 Jun;15(2):79-84.
124. Wood JK, Walker F. Eosinophilic granuloma of lymph nodes. Histopathology. 1977 Jul;1(4):315-6. doi: 10.1111/j.1365-2559.1977.tb01669.x.
125. Woodford NW, Dowling JP, Judson R. Langerhans cell histiocytosis and metastatic lymphoepithelioma-like carcinoma of the parotid. Histopathology. 1997 Oct;31(4):389-90.
126. Wu JH, Lu MY, Lin KH, Jou ST, Lin DT. Development of acute lymphoblastic leukemia in a child after treatment of Langerhans cell histiocytosis: report of one case. Acta Paediatr Taiwan. 1999 Nov-Dec;40(6):441-2.
127. Yagita K, Iwai M, Yagita-Toguri M, Kimura H, Taniwaki M, Misawa S, Okanoue T, Kashima K, Tsuchihashi Y. Langerhans cell histiocytosis of an adult with tumors in liver and spleen. Hepatogastroenterology. 2001 Mar-Apr;48(38):581-4.
128. Yamashita H, Nagayama M, Kawashima M, Hidano A, Yamada O, Mizoguchi H. Langerhans-cell histiocytosis in an adult patient with multiple myeloma. Clin Exp Dermatol. 1992 Jul;17(4):275-8. doi: 10.1111/j.1365-2230.1992.tb02167.x.
129. Yohe SL, Chenault CB, Torlakovic EE, Asplund SL, McKenna RW. Langerhans cell histiocytosis in acute leukemias of ambiguous or myeloid lineage in adult patients: support for a possible clonal relationship. Mod Pathol. 2014 May;27(5):651-6. doi: 10.1038/modpathol.2013.181.
130. Yokokawa Y, Taki T, Chinen Y, Kobayashi S, Nagoshi H, Akiyama M, Morimoto A, Ida H, Taniwaki M. Unique clonal relationship between T-cell acute lymphoblastic leukemia and subsequent Langerhans cell histiocytosis with TCR rearrangement and NOTCH1 mutation. Genes Chromosomes Cancer. 2015 Jul;54(7):409-17. doi: 10.1002/gcc.22252.
131. Yoo JH, Rivera A, Naeini RM, Yedururi S, Bayindir P, Megahead H, Fuller GN, Suh JS, Adesina AM, Hunter JV. Melanotic paraganglioma arising in the temporal horn following Langerhans cell histiocytosis. Pediatr Radiol. 2008 May;38(5):571-4. doi: 10.1007/s00247-007-0734-4.

Table 3 shows aggregate data with details of the observed leukemias and myeloproliferative disorders (Table 3A), lymphomas (Table 3B), solid tumors (Table 3C) by age at LCH diagnosis and timing of occurrence of the AM.

Details of the 19 cases in which LCH and the corresponding AM occurred in a different age group (childhood or adulthood) are reported in Table 4. In particular, 17 LCH cases during childhood had the AM as adults and 2 LCH cases during adulthood had the AM during childhood.

**Table 2:** Details of LCH organ system involvement by age at LCH.

	Age at LCH diagnosis		
Disease category	≤18 years	>18 years	Total
**Single-system**	**53 (45.7)**^1^	**125 (81.2)**^2^	**178 (65.9)**
Bone	39	7	46
Skin	9	21	30
Lymph	2	34	36
Lungs	2	52	54
Thyroid	-	4	4
Urinary tract-Genital organs	-	2	2
Liver	-	1	1
Oral Soft tissue	-	1	1
Gastro-intestinal	-	1	1
Other	1	2	3
**Multi-system**	**61 (52.6)**^3^	**29 (18.8)**^4^	**90 (33.3)**
**Total systems**	209	71	280
Bone	47	8	55
Skin	42	14	56
Lymph	24	13	37
Liver	21	2	23
Lungs	19	13	32
Bone marrow	16	7	23
Spleen	15	4	19
Oral soft tissue	7	2	9
Other soft tissue	5	3	8
CNS	4	2	6
Ear	3	-	3
Gastro-intestinal	1	-	1
Other	5	3	8
**Unknown**	**2 (1.7)**	**0**	**2 (0.7)**
**Total LCH**	**116**	**154**	**270**

1,2 1 case of diabetes insipidus.

3 12 cases of diabetes insipidus.

4 2 cases of diabetes insipidus.

**Table 3(A):** Association of LCH with leukemias and myeloproliferative disorders by age at LCH diagnosis and occurrence of AM with respect to LCH diagnosis.

	Age at LCH diagnosis							
	≤18 years				>18 years			
	Associated Malignancy				Associated Malignancy			
	precedes	concurrent	follows	Total	precedes	concurrent	follows	Total
**Leukemias and myeloproliferative disorders**								
**ALL**	**19**	**-**	**7**	**26**	**2**	**-**	**-**	**2**
*B-ALL*	5	-	2	7	1	-	-	1
*T-ALL*	12	-	3	15	-	-	-	-
*Unspecified*	2	-	2	4	1	-	-	1
**AML**	**-**	**1**	**24**	**25**	**1**	**7**	**9**	**17**
*FAB M1*	-	-	5	5	1	1	-	2
*FAB M2*		-	2	2	-	1	1	2
*FAB M3*	-	-	9	9	-	-	-	-
*FAB M4*	-	1	1	2	-	1	4	5
*FAB M4/M5*	-	-	1	1	-	-		-
*FAB M5*	-	-	2	2	-	-	4	4
*FAB M7*	-	-	1	1	-	-	-	-
*Acute basophilic*	-	-	-	-	-	1	-	1
*Unspecified*	-	-	3	3	-	3	-	3
**Other**	**-**	**4**	**3**	**7**	**3**	**10**	**5**	**18+1**
*Undifferentiated acute leukemia*	-	-	-	-	-	1	-	1
*Mixed lineage acute leukemia*	-	-	-	-	-	1	-	1
*CLL*	-	-	-	-	-	1	-	1
*Myeloid sarcoma*	-	-	-	-	-	2	-	2
*CMML*	-	-	-	-	-	3	4	7
*JCML*			1	1	-	-	-	-
*Polycythemia* *vera*	-	-	-	-	-	1	-	1
*Essential trombocytemia*	-	-	-	-	1	-		1
*MDS*		4	2	6	2	1	1	4
**Total**	**19**	**5**	**34**	**58**	**6**	**17**	**14**	**37+1**

**Legend**: PTLD: Post-transplant lymphoproliferative disorder; ALL: acute lymphoblastic leukemia; AML: acute myeloid leukemia; FAB M1: acute myeloid leukemia without maturation; FAB M2: acute myeloid leukemia with maturation; FAB M3: acute promyelocytic leukemia; FAB M4: acute myelomonocytic leukemia; FAB M5: acute monocytic leukemia; FAB M7: acute megakaryoblastic leukemia; MDS: myelodysplastic syndrome; JCML: juvenile chronic myelocytic leukemia; CMML: chronic myelomonocytic leukemia; CLL: chronic lymphocytic leukemia; +n: number of malignancies subsequent to the first AM.

**Table 3(B):** Association of LCH with lymphomas by age at LCH diagnosis and occurrence of AM with respect to LCH diagnosis.

	Age at LCH diagnosis							
	≤18 years				>18 years			
	Associated Malignancy				Associated Malignancy			
	precedes	concurrent	follows	Total	precedes	concurrent	follows	Total
**Lymphomas**								
Hodgkin	2	1	4	**7**	12	17	3	**32**
Non-Hodgkin	2	1	4	**7**	6	13	4	**23+2**
*Large B-cell*	*1*	*-*	*3*	*4*	*-*	*3*	*-*	*3*
*Immunoblastic*	*-*	*-*	*-*	*-*	*-*	*1*	*-*	*1*
*Follicular*	*-*	*-*	*-*	*-*	*3*	*1*	*1*	*5*
*Follicular grade 2*	*-*	*-*	*-*	*-*	*-*	*3*	*-*	*3*
*Follicular grade 1*	*1*	*-*	*-*	*1*	*-*	*-*	*-*	*-*
*Lymphoblastic*	*-*	*-*	*-*	*-*	*-*	*2*	*-*	*2*
*T-cell lymphoblastic*	*-*	*1*	*-*	*1*	*-*	*3*	*2*	*5*
*Multiple myeloma*	*-*	*-*	*-*	*-*	*2*	*-*	*-*	*2*
*Unspecified*	*-*	*-*	*1*	*1*	*1*	*-*	*1*	*2*
Histiocytic sarcoma	-	-	-	**+1**	-	-	1	**1+1**
PTLD	-	-	1	**1**	-	-	-	**-**
**Total**	**4**	**2**	**9**	**15+1**	**18**	**30**	**8**	**56+3**

**Legend**:

PTLD: Post transplant proliferative disorder.

+n: number of malignancies subsequent to the first AM.

**Table 3(C):** Association of LCH with solid tumors by age at LCH diagnosis and occurrence of AM with respect to LCH diagnosis.

	Age at LCH diagnosis							
	≤18 years				>18 years			
	Associated Malignancy				Associated Malignancy			
Site/type of solid tumor	precedes	concurrent	follows	Total	precedes	concurrent	follows	Total
**Lung**	**-**	**-**	**1**	**1**	**5**	**9**	**9**	**23+3**
*Carcinoma*	*-*	*-*	*1*	*1*	*3*	*2*	*5*	*10*
*Adenocarcinoma*	*-*	*-*	*-*	*-*	*2*	*4*	*4*	*10*
*Bronchiolo-alveolar carcinoma*	*-*	*-*	*-*	*-*	*-*	*1*	*-*	*1*
*Carcinoid tumor*	*-*	*-*	*-*	*-*	*-*	*1*	*-*	*1*
*Langerhans cell sarcoma*	*-*	*-*	*-*	*-*	*-*	*1*	*-*	*1*
**CNS**	**5**	**1**	**8**	**14**	**1**	**-**	**-**	**1**
*PNET*	*2*	*-*	*-*	*2*	*-*	*-*	*-*	*-*
*Astrocytoma*	*-*	*-*	*1*	*1*	*-*	*-*	*-*	*-*
*Germ cell tumor*	*1*	*-*	*-*	*1*	*-*	*-*	*-*	*-*
*Brain stem anaplastic tumor*	*-*	*1*	*-*	*1*	*-*	*-*	*-*	*-*
*Ependymoma*	*-*	*-*	*3*	*3*	*-*		*-*	*-*
*Medulloblastoma*	*2*	*-*	*1*	*3*	*-*	*-*	*-*	*-*
*Meningioma*	*-*	*-*	*2*	*2*	*-*	*-*	*-*	*-*
*Oligodendroglioma*	*-*	*-*	*-*	*-*	*1*	*-*	*-*	*1*
*Paraganglioma*	*-*	*-*	*1*	*1*	*-*	*-*	*-*	*-*
**Skin**	**-**	**-**	**4**	**4**	**3**	**2**	**-**	**5+2**
*Basal cell carcinoma*	*-*	*-*	*4*	*4*	*1*	*2*	*-*	*3*
*Melanoma*	*-*	*-*	*-*	*-*	*2*	*-*	*-*	*2*
**Thyroid carcinoma**	**-**	**-**	**2**	**2+1**	**3**	**4**	**-**	**7**
**Breast carcinoma**	**-**	**-**	**1**	**1**	**6**	**-**	**-**	**6+1**
**Retinoblastoma**	**3**	**1**	**2**	**6**	**-**	**-**	**-**	**-**
**Bone**	**1**	**-**	**6**	**7**	**-**	**-**	**-**	**-**
*Osteosarcoma*	*-*	*-*	*3*	*3*	*-*	*-*	*-*	*-*
*Ewing sarcoma*	*1*	*-*	*2*	*3*	*-*	*-*	*-*	*-*
*Gigant cell tumor*	*-*	*-*	*1*	*1*	*-*	*-*	*-*	*-*
**Male genital system**	**-**	**-**	**-**	**-**	**4**	**-**	**-**	**4+2**
*Testicular teratoma*	*-*	*-*	*-*	*-*	*1*	*-*	*-*	*1*
*Testicular embrional carcinoma*	*-*	*-*	*-*	*-*	*1*	*-*	*-*	*1*
*Seminoma*	*-*	*-*	*-*	*-*	*1*	*-*	*-*	*1*
*Testicular germ cell tumor*	*-*	*-*	*-*	*-*	*1*	*-*	*-*	*1*
**Neuroblastoma**	**2**	**3**	**-**	**5**	**-**	**-**	**-**	**-**
**Gastro intestinal**	**-**	**-**	**-**	**-**	**1**	**2**	**-**	**3**
*Colon adenocarcinoma*	*-*	*-*	*-*	**-**	*1*	*1*	*-*	*2*
*Gastric carcinoma*	*-*	*-*	*-*	**-**	*-*	*1*	*-*	*1*
**Urinary tract**	**-**	**1**	**-**	**1**	**1**	**2**	**1**	**4+1**
*Renal carcinoma*	*-*	*-*	*-*	*-*	*-*	*1*	*1*	*2*
*Wilms tumor*	*-*	*1*	*-*	*1*	*-*	*-*	*-*	*-*
*Bladder carcinoma*	*-*	*-*	*-*	*-*	*1*	*-*	*-*	*1*
*Papillary urothelial carcinoma*	*-*	*-*	*-*	*-*	*-*	*1*	*-*	*1*
**Female genital system**	**-**	**-**	**-**	**-**	**1**	**-**	**1**	**2**
*Ovary dysgerminoma*	*-*	*-*	*-*	*-*	*1*	*-*	*-*	*1*
*Vulvar squamous cell carcinoma*	*-*	*-*	*-*	*-*	*-*	*-*	*1*	*1*
**Liver**	**-**	**-**	**2**	**2**	**-**	**-**	**-**	**-**
*Apudoma*	*-*	*-*	*1*	*1*	*-*	*-*	*-*	**-**
*Hepatocellular carcinoma*	*-*	*-*	*1*	*1*	*-*	*-*	*-*	**-**
**Pancreatic cancer**	**-**	**-**	**-**	**-**	**-**	**-**	**1**	**1**
**Parotid carcinoma**	**-**	**-**	**-**	**-**	**-**	**1**	**-**	**1**
**Malignant fibrous histiocytoma**	**-**	**-**	**-**	**-**	**-**	**-**	**1**	**1**
**Carcinoma of the tongue**	**-**	**-**	**-**	**-**	**-**	**1**	**-**	**1**
**Undefined carcinoma**	**-**	**-**	**-**	**-**	**-**	**1**	**1**	**2**
**Total**	**11**	**6**	**26**	**43+1**	**25**	**22**	**14**	**61+9**

**Legend:**

CNS: Central nervous system.

PNET: Primitive neuro-ectodermal tumor.

+n: number of malignancies subsequent to the first AM.

**Table 4:** LCH diagnosis and occurrence of AM in different groups of age.

LCH during childhood and AM during adulthood		
Associated Malignancy	N	Interval (years) of AM occurrence after LCH diagnosis
**AML**	**2**	
FAB M2	1	25
FAB M5	1	14
**Lymphomas**	**2**	
Hodgkin	1	7
Non-Hodgkin	1	20
**Solid tumors**	**13**	
Bone	2	11; 22
Breast	1	27
CNS	3	13; 21; 13
Liver	2	18; 19
Lung	1	14
Thyroid	1	17
Skin	3	12; 25; 36

LCH during adulthood and AM during childhood		

Associated Malignancy	N	Interval (years) of AM occurrence before LCH diagnosis

**Lymphomas**	**2**	
Hodgkin	2	-7; -6

## 2 Experimental Design, Materials and Methods

This dataset was initiated in 1991 by R. Maarten Egeler that invited HS members to report any patient of any age they were currently treating, or had previously treated with LCH and another malignancy which may have occurred before, concurrently or after the LCH diagnosis. Further updates were made through periodic review of the scientific literature and of abstracts of the annual HS meetings or other international meetings attended by one of the authors. At the end of 2001, the responsibility of the project was transferred to R. Haupt and periodic searches through the PubMed data base were continued until 2015 [search terms: ("Histiocytosis, Langerhans-Cell"[Mesh] or "Langerhans cell histiocytosis"[tiab] or histiocytosis[tiab] or “eosinophilic granuloma”[tiab]) and ("neoplasms"[Majr] or leukemia[tiab] or lymphoma[tiab]) Filters: English]. The titles/abstracts of all studies identified by the search were screened by one reviewer (RH). The full texts of the potentially eligible studies were then obtained, and reviewers (RH/BC/FB) checked whether the articles fully complied with the inclusion criteria. For each reported case of LCH-AM occurrence a CRF was completed with all the data available in the manuscript; uncertain cases were identified by each reviewer and then discussed within the team. Since the same subject might have been reported in different case series, a further screening was made to identify duplicates after controlling for the pattern of association, the reporting institution and clinical details. Additionally, other articles, if any, included among references of selected manuscripts but not identified by the literature search, were screened by one reviewer (RH).

Patients were further stratified based on their age at LCH diagnosis with 18 years being the cut-off between children and adults. The associated malignancies were identified based on their histopathological report, as described in the manuscripts and/or in CRFs and original reports sent on voluntary basis, and further stratified in 4 categories: i) acute lymphoblastic leukemia; ii) acute myeloid leukemia, other leukemias and myeloproliferative disorders; iii) lymphomas (Hodgkin and non Hodgkin); iv) solid tumors.

Because of the only descriptive nature of this dataset, statistics were limited to calculating the interval between LCH and AM diagnosis and absolute frequencies and percentages by using Stata (StataCorp. Stata Statistical Software, Release 16.1 College Station, TX, Stata Corporation, 2019).

## Ethics Statements

No formal approval by the Ethics Review Board was necessary at the moment of the project set-up (1991) and most of the dataset is based on literature review; for cases identified only through the study CRFs the authorization by the reporting physician to use the data was considered implicit with the voluntary transmission of data, we thus do not have any informed consent by the study subjects. According to guidelines by the General Data Protection Authority for secondary use of health data, it is possible to use data for research purposes “ … if the researcher can show that pseudonymisation – or another aggregated data process – has been used so the researchers cannot identify the individual, and therefore cannot contact them to obtain consent”. We believe that this statement applies to our dataset. The LCH-AM database is stored in a secure server at the Gaslini Institute, Genova, Italy.

## CRediT Author Statement

**Francesca Bagnasco:** Data curation, Software, Formal analysis, Visualization, Investigation, Writing – original draft preparation, Writing – review & editing; **Stefanie-Yvonne Zimmermann:** Investigation, Writing – original draft preparation, Writing – review & editing; **R. Maarten Egeler:** Conceptualization, Methodology, Resources, Writing – review & editing; **Vasanta Rao Nanduri:** Resources, Writing – review & editing; **Bruna Cammarata:** Resources, Investigation, Writing – review & editing; **Jean Donadieu:** Resources, Writing – review & editing; **Thomas Lehrnbecher:** Methodology, Resources, Writing – original draft preparation, Writing – review & editing, Supervision; **Riccardo Haupt:** Conceptualization, Methodology, Resources, Investigation, Writing – original draft preparation, Writing – review & editing, Supervision, Project administration.

## Acknowledgments

We thanks the following colleagues for their contribution in data retrieval:

• Aricò Maurizio, Oncoematologia Pediatrica, Ospedale dei bambini, Palermo, Italy.
• Bailey Simon, The Newcastle upon Tyne Hospitals, England, UK.
• Campbell Patrick K., Department of Oncology, St Jude Children's Research Hospital, Memphis, USA.
• Carroll Michelle, Children's National Medical Center, USA.
• Elfride Thiem, Children Cancer Research Institute, Vienna, Austria.
• Giona Fiorina, Haematology, Department of Translational and Precision Medicine, Sapienza University of Rome, Italy.
• Girschikofsky Michael, Elisabethinen Hospital, Linz, Austria.
• Grant Ronald M., Department of Hematology/Oncology, The Hospital for Sick Children, Toronto, Canada.
• McClain Kenneth L., Pediatrics and Hematology/Oncology, Texas Children's Cancer Center, USA.
• Nichols Kim, Department of Pediatric Hematology and Oncology, The Children's Hospital of Philadelphia, USA.
• van den Bos Cor, Department of Pediatric Oncology, Princess Maxima Center for pediatric Oncology, Utrecht, The Netherlands.

This research was partially supported by the Italian Minstry of Health - Ricerca Corrente 2022 - no Grant ID applicable.

## Supplementary materials

LCH-AM CRF.pdf: Case report form for registering the study subjects.

## Declaration of Competing Interest

The authors declare that they have no known competing financial interests or personal relationships that could have appeared to influence the work reported in this paper.

## Data Availability

LCH and malignancy association (Original data) (Mendeley Data).

## Appendix Supplementary materials

Image, application 1
