# Supplementary material for: The international dataset on the association between Langerhans Cell Histiocytosis and other malignancies
Source: Data Brief. 2022 Sep 15;45:108604. doi: 10.1016/j.dib.2022.108604 (PMC9519425; doi:10.1016/j.dib.2022.108604)
Supplement: Supplementary file 1 [file mmc1.pdf]

LANGERHANS CELL HISTIOCYTOSIS - MALIGNANCY STUDY

Patient's data

ID code ☐☐☐☐☐ Center ____________________ Country ______________

Sex: M F (**M**=Male; **F**=Female)

Ethnicity: ☐ (**1=**Asian; **2=**White; **3=**Black; **4=**Hispanic; **5=**Other) If Other, specify: ______________

Langerhans cell histiocytosis ☐

Age at LCH diagnosis (yrs) ☐☐☐☐☐

Date of LCH diagnosis: ☐☐☐☐☐☐ (dd mm yy)

Extension: ☐
1 = Single system / Single site
2 = Single system / Multiple site
3 = Multi system

1. Systems involved (check all that apply)

Bone ☐ Skin ☐ Lymph ☐ Diabetes Insipidus ☐ Liver ☐

Spleen ☐ Lungs ☐ Bone Marrow ☐ Oral Soft Tissue ☐ GI ☐ CNS ☐

Other: ☐ ______________

System biopsed? N Y if yes, specify system(s):

System ________ ***System*** ________ ***System*** ________ ***System*** ________

2. Histology (please, enclose histopathology forms/electron microscopy results/results of monoclonal antibodies)

	Light microscopy	S100	ATP-ase	Pean Lect	Alfa-D-Mann	Birbek	OKT6/CD1a
positive	☐	☐	☐	☐	☐	☐	☐
negative	☐	☐	☐	☐	☐	☐	☐
not done	☐	☐	☐	☐	☐	☐	☐

3. Treatment (check all that apply)

Tratment? N Y if yes, specify:

Surgery/Curettage N Y

Radiation N Y if yes, specify:

dosage applied (cGy): ☐☐☐☐ Site / Field ______________
dosage applied (cGy): ☐☐☐☐ Site / Field ______________
dosage applied (cGy): ☐☐☐☐ Site / Field ______________

Steroids: N Y if yes, specify:

Oral ☐ IV ☐ Local ☐ Intralesional ☐

Chemotherapy: N Y if yes, specify:

VBL ☐ VCR ☐ VP16 ☐

Chlorambucil ☐ MTX ☐ Others ______________

Immunotherapy: N Y if yes, specify:

alfa-IFN ☐ Cyclosporine ☐ Others ______________

Starting date therapy: ☐☐☐☐☐☐ (dd mm yy) Ending date last therapy: ☐☐☐☐☐☐ (dd mm yy)

4. Duration of LCH disease and follow-up

Remission [N] [Y] Date of 1st remission: [| | | | |] (dd mm yy)

Relapse [N] [Y] N° relapses: [|] Date of 1st relapse: [| | | | |] (dd mm yy)

Associated malignancy

Date of malignancy diagnosis: [| | | | |] (dd mm yy) Age at malignancy (yrs) [| | | |]

5. Diagnosis ______________________________

Histology / Cytogenetics / Cell markers ______________________________
(Please, enclose documentation)

Stage / Extent of disease / Site of disease: ______________________________

Did the malignancy occur within previous radiation port?

[ ] Yes [ ] No [ ] Don't know [ ] Not Irradiated [ ] Not Applicable (if malignancy preceded LCH)

6. Treatment (check all that apply)

Tratment? [N] [Y] If yes, specify

Surgery [N] [Y]

Radiation [N] [Y] If yes, specify

dosage applied (cGy): [| | |] Site / Field ______________________________

dosage applied (cGy): [| | |] Site / Field ______________________________

dosage applied (cGy): [| | |] Site / Field ______________________________

Chemotherapy [N] [Y] If yes, specify

specify: ______________________________

Starting date therapy: [| | | | |] (dd mm yy) Ending date last therapy: [| | | | |] (dd mm yy)

7. Duration of associated malignancy and follow-up

Remission [N] [Y] Date of 1st remission: [| | | | |] (dd mm yy)

Relapse [N] [Y] N° relapses: [|] Date of 1st relapse: [| | | | |] (dd mm yy)

Current status

8. Current status

Date of last information: [| | | | |] (dd mm yy) Status: [A] [D] (**A**=Alive; **D**=Dead)

If alive: Alive with LCH [ ] Alive with malignancy [ ]
(check all that apply)

If dead: Died with LCH [ ] Died with malignancy [ ] Died of intercurrent disease [ ]
(check all that apply)

Cause of death: ______________________________

Notes ____

____

____

Date: ____________ **Signature:** ________________________
